# Intradiscal transplantation of synovial mesenchymal stem cells prevents intervertebral disc degeneration through suppression of matrix metalloproteinase-related genes in nucleus pulposus cells in rabbits

**DOI:** 10.1186/ar3182

**Published:** 2010-11-05

**Authors:** Takashi Miyamoto, Takeshi Muneta, Takashi Tabuchi, Kenji Matsumoto, Hirohisa Saito, Kunikazu Tsuji, Ichiro Sekiya

**Affiliations:** 1 Section of Orthopaedic Surgery, Tokyo Medical and Dental University, 1-5-45 Yushima, Bunkyo-ku, Tokyo 113-8519, Japan; 2 Global Center of Excellence Program; International Research Center for Molecular Science in Tooth and Bone Diseases, Tokyo Medical and Dental University, 1-5-45 Yushima, Bunkyo-ku, Tokyo 113-8519, Japan; 3 Medical Satellite Yaesu Clinic, 1-5-9 Yaesu, Chuo-ku, Tokyo 103-0028, Japan; 4 Department of Allergy and Immunology, National Research Institute for Child Health and Development, 2-10-1 Okura, Setagaya-ku, Tokyo 157-8535, Japan; 5 Section of Cartilage Regeneration, Tokyo Medical and Dental University, 1-5-45 Yushima, Bunkyo-ku, Tokyo 113-8519, Japan

## Abstract

**Introduction:**

Synovial mesenchymal stem cells (MSCs) have high proliferative and chondrogenic potentials, and MSCs transplanted into the articular cartilage defect produce abundant extracellular matrix. Because of similarities between the articular cartilage and the intervertebral disc cartilage, synovial MSCs are a potential cell source for disc regeneration. Here, we examined the effect of intradiscal transplantation of synovial MSCs after aspiration of nucleus pulposus in rabbits.

**Methods:**

The nucleus pulposus tissues of rabbit's intervertebral discs were aspirated to induce disc degeneration, and allogenic synovial MSCs were transplanted. At 2, 4, 6, 8, 16, 24 weeks postoperatively, we evaluated with imaging analyses such as X-ray and magnetic resonance imaging (MRI), and histological analysis. To investigate interaction between synovial MSCs and nucleus pulposus cells, human synovial MSCs and rat nucleus pulposus cells were co-cultured, and species specific microarray were performed.

**Results:**

The existence of transplanted cells labeled with DiI or derived from green fluorescent protein (GFP)-expressing transgenic rabbits was confirmed up until 24 weeks. X-ray analyses demonstrated that intervertebral disc height in the MSC group remained higher than that in the degeneration group. T2 weighted MR imaging showed higher signal intensity of nucleus pulposus in the MSC group. Immunohistological analyses revealed higher expression of type II collagen around nucleus pulposus cells in the MSC group compared with even that of the normal group. In co-culture of rat nucleus pulposus cells and human synovial MSCs, species specific microarray revealed that gene profiles of nucleus pulposus were altered markedly with suppression of genes relating matrix degradative enzymes and inflammatory cytokines.

**Conclusions:**

Synovial MSCs injected into the nucleus pulposus space promoted synthesis of the remaining nucleus pulposus cells to type II collagen and inhibition of expressions of degradative enzymes and inflammatory cytokines, resulting in maintaining the structure of the intervertebral disc being maintained.

## Introduction

Intervertebral discs lie between adjacent vertebrae in the spine and are composed of three major structures called nucleus pulposus, annulus fibrosus, and cartilage end plates [1]. The nucleus pulposus of normal disc includes sparse chondrocytes surrounded by extracellular matrix which mainly consist of type II collagen and proteoglycan. It functions as a shock absorber against mechanical load due to its highly hydrophilic structure. Intervertebral disc degeneration accompanies aging, and it causes low back pain [2,3]. To regenerate intervertebral discs, various approaches applying cytokines [4,5], gene transfection [6], and nucleus pulposus cells [7] have been attempted in animal models. Some reports have demonstrated that transplantation of bone marrow mesenchymal stem cells (MSCs) delayed degeneration of the nucleus pulposus [8-10].

An increasing number of reports have shown that MSCs can be isolated from other various mesenchymal tissues other than bone marrow, and that their similarities as MSCs and the specificities dependent of their MSC source are emerging [11-13]. Our comparative *in vivo *study showed that bone marrow MSCs and synovial MSCs produced a higher amount of cartilage matrix than adipose MSCs and muscle MSCs after transplantation into articular cartilage defect of the knee in rabbits [14]. We also demonstrated that synovial MSCs expanded faster than bone marrow MSCs when cultured with 10% human autologous serum [15]. Synovial MSCs and bone marrow MSCs have a similar chondrogenic potential, but synovial MSCs are more useful from the standpoint of yield when cultured with human autologous serum.

Histologically and biochemically, some similarities exist between the nucleus pulposus and the articular cartilage. In this study, we investigated whether intradiscal transplantation of synovial MSCs delayed disc degeneration in a rabbit model. MSCs labeled with DiI or derived from green fluorescent protein (GFP) expressing transgenic rabbit [16] were used for tracking of transplanted cells. Furthermore, human synovial MSCs and rat nucleus pulposus cells were co-cultured *in vitro*, and their interaction was clarified by a species specific microarray system. Finally, we demonstrated the effectiveness and limitations of this method and advocated a possible mechanism to prevent intervertebral disc degeneration in a rabbit model.

## Materials and methods

### Cell isolation and culture

This study was approved by the Animal Experimentation Committee of Tokyo Medical and Dental University. Wild type Japanese white rabbits and GFP transgenic rabbits [16] (Kitayama Labes Co., Ltd., Nagano, Japan) were anesthetized with an intramuscular injection of 25 mg/kg ketamine hydrochloride and 150 μg/kg medetomidine hydrochloride. Synovium was harvested aseptically from knee joints of the rabbits, and bone marrow was obtained from their femurs by flushing with Hanks' balanced salt solution (Invitrogen, Carlsbad, CA, USA).

The harvested synovium was digested in a 3 mg/ml collagenase type V (Sigma-Aldrich Co., St. Louis, MO, USA) in α-minimal essential medium (αMEM) (Invitrogen) for three hours at 37°C. The digested tissues were filtered through a cell strainer (Becton, Dickinson and Company, Franklin Lakes, NJ, USA) with 70-μm pore size. The obtained cells were seeded at 5 × 10^4 ^cells/cm^2 ^in 145-cm^2 ^culture dishes (Nalge Nunc International, Rochester, NY, USA) and cultured with complete medium, αMEM containing 10% fetal bovine serum (FBS), 100 units/ml penicillin, 100 μg/ml streptomycin, and 250 ng/ml amphotericin B. The medium was replaced to remove nonadherent cells two days later. After being cultured for seven days, the cells were harvested with 0.25% trypsin-EDTA (Invitrogen) and cryopreserved at 1 × 10^6 ^cells/ml in αMEM with 5% dimethylsulfoxide (Wako, Osaka, Japan) and 10% FBS.

The harvested bone marrow was filtered through a cell strainer with 70-μm pore size and plated in 145-cm^2 ^culture dishes with the medium described above and then incubated at 37°C with 5% humidified CO_2_. The medium was replaced the next day. After being cultured for 14 days, the cells were harvested with 0.25% trypsin-EDTA, replated in 145-cm^2 ^culture dishes, and cultured as passage 1. Passage 1 cells were harvested and cryopreserved after the culture for 14 days.

The frozen cells from synovium and bone marrow were thawed, plated at 3 × 10^3 ^cells/cm^2 ^in 145-cm^2 ^culture dishes, and incubated for five days. Harvested cells derived from wild type rabbits with 0.25% trypsin-EDTA were resuspended at 1 × 10^6 ^cells/ml in αMEM, and a DiI (Molecular Probes, Eugene, OR, USA) fluorescent lipophilic tracer was added at 5 μl/ml in αMEM. After incubation for 20 minutes at 37°C with 5% humidified CO_2_, the cells were centrifuged at 450 *g *for five minutes and washed twice with PBS. The obtained cells were used for further analyses.

### Colony-forming unit assay

One thousand cells were plated in 60-cm^2 ^dishes, cultured in complete medium for 14 days, and stained with 0.5% Crystal Violet in methanol for five minutes.

### *In vitro *differentiation assay

Five hundred cells from rabbit synovium were plated in 60-cm^2 ^dishes and cultured in complete medium for 14 days. For adipogenesis, the medium was then switched to adipogenic medium that consisted of complete medium supplemented with 10^-7 ^M dexamethasone, 0.5 mM isobutylmethylxanthine, and 100 μM indomethacin. After four days, the adipogenic cultures were stained with 0.3% Oil Red-O solution. For calcification, the medium was then switched to calcification medium that consisted of complete medium supplemented with 10^-9 ^M dexamethasone, 10 mM β-glycerol phosphate, and 50 μg/ml ascorbate-2-phosphates for an additional six weeks. These dishes were stained with 0.5% Alizarin Red solution. For chondrogenesis, 2 × 10^5 ^cells were plated in a 15 ml polypropylene tube (BD Falcon, Bedford, MA, USA) and pelleted by centrifugation at 450 *g *for 10 minutes. The pellets were cultured for 21 days in chondrogenic medium which contained 1,000 ng/ml bone morphogenetic protein 7 (Stryker Biotech, Boston, MA, USA), 10 ng/ml transforming growth factor-β3 (R&D Systems Inc., Minneapolis, MN, USA), and 100 nM dexamethasone. For histological analysis, the pellets were embedded in paraffin, cut into 5-μm sections, and stained with 1% Toluidine Blue.

### *In vivo *transplantation

Mature female Japanese white rabbits weighing an average of 3.0 kg were anesthetized as mentioned above. The anterior surface of the lumbar spine was exposed through the anterolateral approach, and then L3 to L4, L4 to L5, and L5 to L6 intervertebral discs were identified. Nucleus pulposus tissues at L3 to L4 and L5 to L6 discs were aspirated using a 21-gauge needle on a 10 ml syringe to induce disc degeneration [10]. Then, L3 to L4 discs were untreated and referred to as the "degeneration group." L5 to L6 discs were injected with 100 μl of 1 × 10^7 ^allogenic MSCs/ml in PBS using a 27-gauge needle immediately after the nucleus pulposus aspiration, and referred to as the "MSC group." L4 to L5 discs were approached but not treated and referred to as the "normal group." After the operation, all rabbits were allowed to move in a cage freely. At 2, 4, 6, 8, 16, 24 weeks postoperatively, the rabbits were sacrificed with an overdose of sodium pentobarbital, and the lumbar spine was harvested.

### Imaging analysis

Radiographs were taken immediately after harvest of the lumbar spine using X-ray equipment (CMB-2; SOFTEX, Kanagawa, Japan). Intervertebral disc height and vertebral body height were measured, and the disc height index (DHI) [17] was calculated. Alterations in the DHI were normalized to the DHI before aspiration of the nucleus pulposus and are indicated as "%DHI".

MR imaging at 3.0T (Achieva; Philips Medical Systems, Andover, MA, USA) was used with an 8-cm diameter surface dual coil. T2-weighted turbo spin-echo images (TE 130 ms, TR 3,200 ms, FOV 140 mm, matrix 320 × 360, slice thickness of 3 mm) of the lumbar spine were obtained at each time point.

### Histology and fluorescent microscopy

The intervertebral discs including the adjacent vertebral bodies were fixed in 4% paraformaldehyde, decalcified with 20% EDTA, dehydrated in a gradient series of ethanol, and embedded in paraffin. Midline sagittal sections of the intervertebral discs were stained with Hematoxylin and Eosin.

For fluorescent microscopy, the nucleus pulposus was harvested, fixed in 4% paraformaldehyde, and transferred to 20% sucrose solution. Specimens were flash-frozen and cut in a cryostat. Sections were mounted on a slide and observed under epifluorescent microscopy.

### Immunohistochemistry

Paraffin-embedded sections were deparaffinized in xylene, rehydrated through graded alcohol, and immersed in PBS. The samples were pretreated with 0.4 mg/ml proteinase K (DAKO, Carpinteria, CA, USA) in Tris-HCl buffer for 15 minutes at room temperature for antigen retrieval. Any residual enzymatic activity was removed by washing with PBS, and nonspecific staining was blocked by preincubation with PBS containing 10% normal horse serum for 20 minutes at room temperature. Mouse monoclonal antibody against human type II collagen (1:1,000 dilution; Daiichi Fine Chemical, Toyama, Japan) was placed on each section for one hour at room temperature. After extensive washing with PBS, the sections were incubated in the secondary antibody of biotinylated horse anti-mouse IgG (Vector Laboratories, Burlingame, CA, USA) for 30 minutes at room temperature. Immunostaining was detected with VECTSTAIN ABC reagent (Vector Laboratories), followed by DAB staining. Counter staining was performed with Mayer-Hematoxylin.

### Co-culture experiments and RNA isolation

Human synovium was obtained during anterior cruciate ligament reconstruction surgery for ligament injury and digested with 3 mg/ml collagenase type V in αMEM for three hours at 37°C. The digested tissues were filtered through a 70-μm pore cell strainer. The obtained cells were incubated with complete medium as human synovial MSCs. Nucleus pulposus tissues were harvested from wild type Wister rat, minced, digested for 30 minutes at 37°C with 0.01% trypsin-EDTA, and filtered through a 70-μm pore cell strainer. Nucleated cells were seeded in 60-cm^2 ^culture dishes (Nalge Nunc International) with αMEM containing 10% FBS.

For co-culture, both passage 2 human synovial MSCs and passage 0 rat nucleus pulposus cells were seeded together in 60-cm^2 ^culture dishes at 3 × 10^3 ^cells/cm^2^, respectively. For the control, only human synovial MSCs or only rat nucleus pulposus cells were seeded at 6 × 10^3 ^cells/cm^2^. After seven days, total RNA was isolated from cultured cells with the RNeasy Total RNA Mini Kit (Qiagen, Valencia, CA, USA).

### Oligonucleotide microarray

Three μg of total RNA from each sample was first reverse transcribed to synthesize the first-strand cDNA using a T7-Oligo(dT) promoter primer by the One-Cycle cDNA Synthesis Kit (Affymetrix, Santa Clara, CA, USA). Following RNase H-mediated second-strand cDNA synthesis, the double-stranded cDNA was purified and served as a template in the subsequent *in vitro *transcription (IVT) reaction. The IVT reaction was carried out in the presence of T7 RNA Polymerase and a biotinylated nucleotide analog/ribonucleotide mix for complementary RNA (cRNA) amplification and biotin labeling using a GeneChip IVT Labeling kit (Affymetrix, Santa Clara, CA, USA). The biotinylated cRNA targets were then cleaned up, fragmented, and hybridized to GeneChip^® ^Rat Genome 230 2.0 probe arrays (Affymetrix) and/or GeneChip^® ^Human U-133 plus 2.0 probe arrays according to the manufacturer's instructions [18].

Data analysis was performed with GeneSpring software version 7.2 (Agilent Technologies, Palo Alto, CA, USA). To normalize the variations in staining intensity among chips, the 'signal' values for all genes on a given chip were divided by the median value for expression of all genes on the chip. To eliminate genes containing only a background signal, genes were selected only if the raw values of the 'signal' were more than the lower limit of the confidence interval. Expression of the gene was judged to be 'present' by the GeneChip Operating Software version 1.4 (Affymetrix). The microarray data were deposited in the Gene Expression Omnibus [19], [GEO:GSE24612]. Genes filtered with this quality criteria were subjected to further analysis.

A hierarchical-clustering analysis was performed using a minimum distance value of 0.001, a separation ratio of 0.5 and the standard definition of the correlation distance. A dendrogram was obtained from hierarchically clustering analysis using average linkage and distance metric equal to one minus the Pearson correlation applied to the microarray data.

### Statistical analysis

To assess differences, two-factor ANOVA and Tukey-Kramer post-hoc tests were used. *P*-values less than 0.05 were considered statistically significant.

## Results

### Characteristics of synovial cells as MSCs

**Figure 1:**
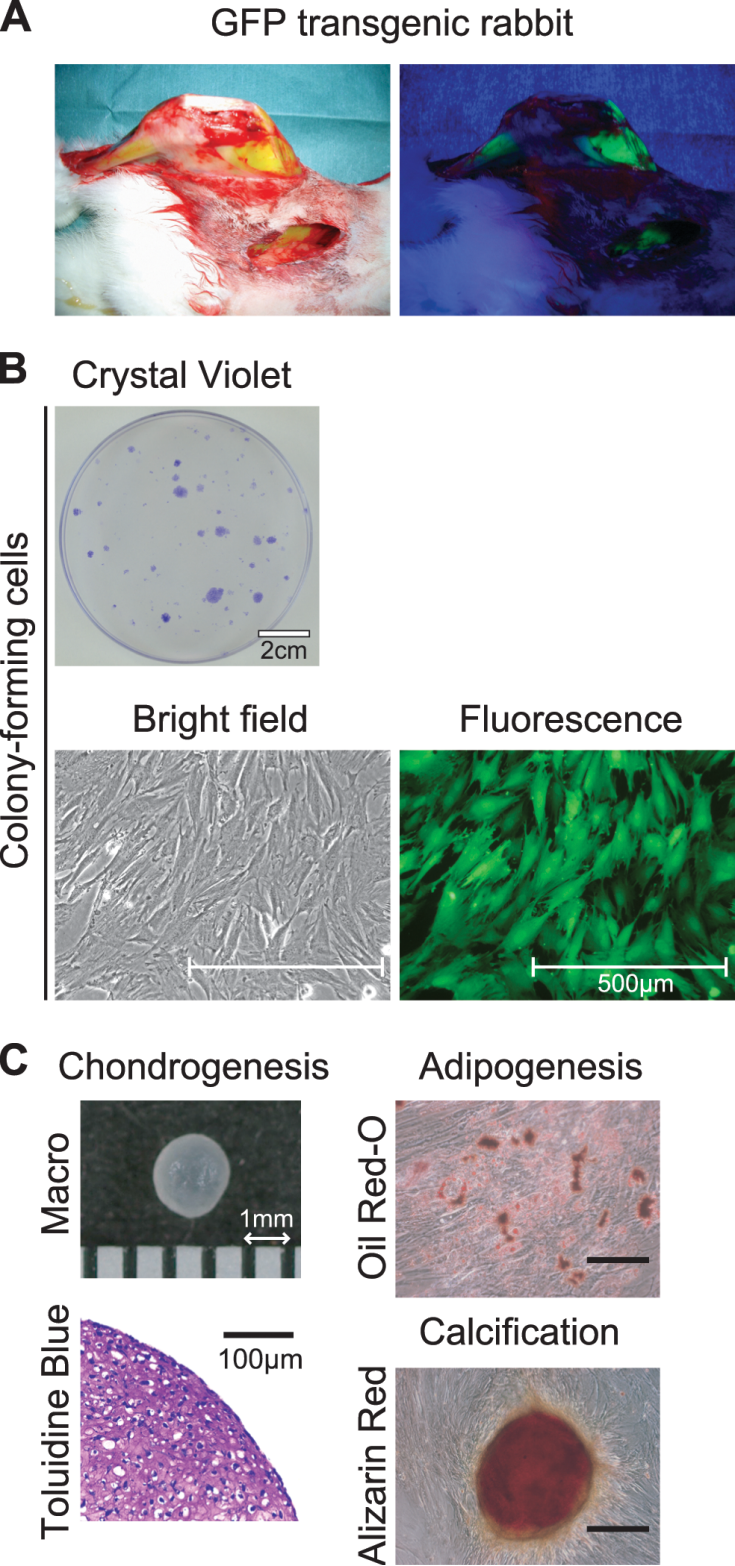
**Cells from rabbit synovium have characteristics of MSCs**. **(a) **Right hindlimb of GFP transgenic rabbit. **(b) **Colony forming cells derived from GFP transgenic rabbit synovium. **(c) **Differentiation potentials.

A GFP rabbit showed green under its skin, especially in its muscles and bones under fluorescence (Figure 1a). Colony forming cells derived from GFP rabbit synovium demonstrated green under fluorescence (Figure 1b). These cells differentiated into chondrocytes and adipocytes, and were calcified when cultured in the appropriate differentiation medium (Figure 1c). As MSCs are defined by adherence to plastic and trilineage differentiation [20], our results indicate that the rabbit synovium-derived cells had characteristics of MSCs.

### Existence of transplanted MSCs

**Figure 2:**
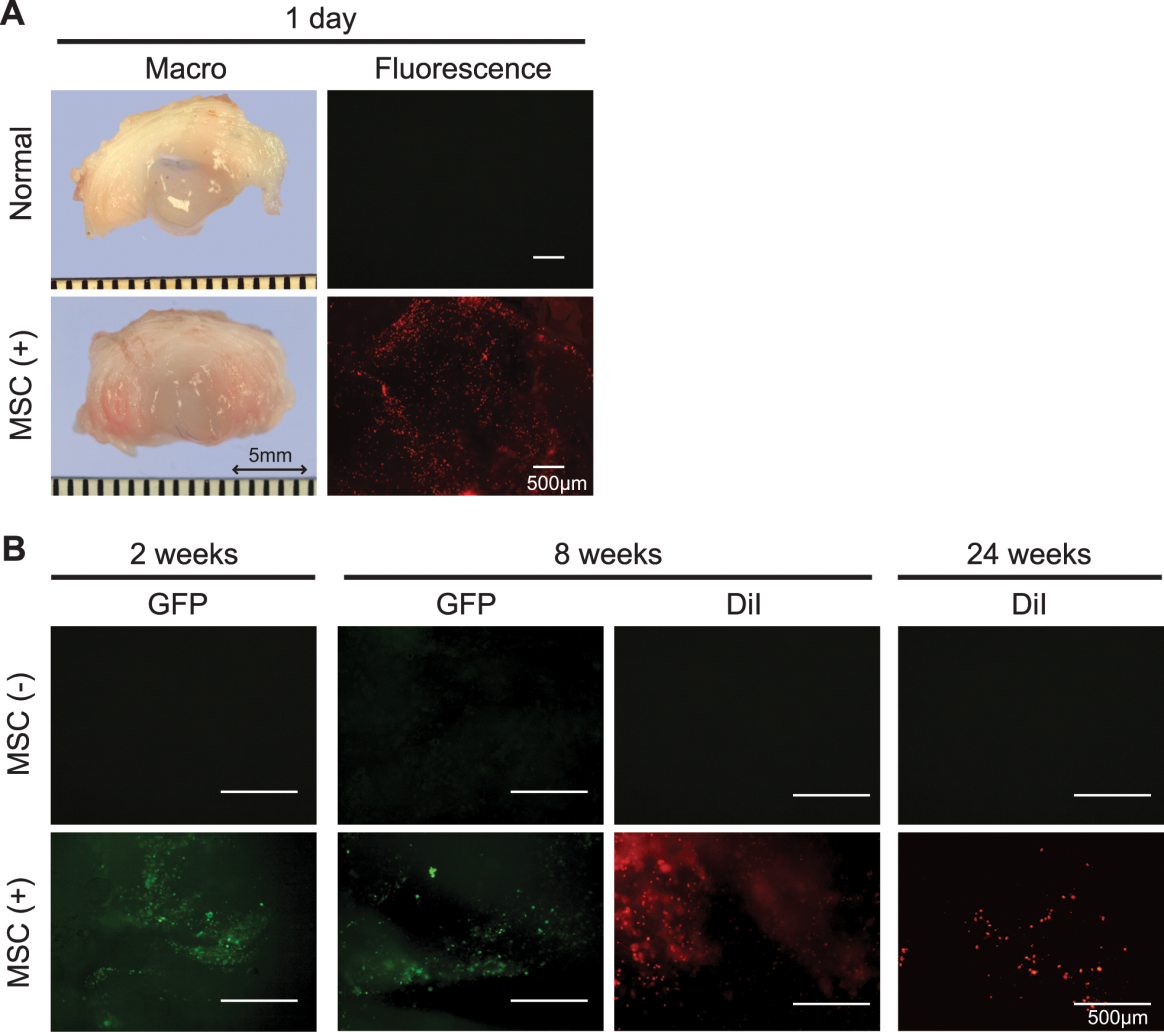
**Intradiscally injected MSCs remain in the nucleus pulposus at 24 weeks**. **(a) **Disc in normal condition and one day after intradiscal injection of DiI-labeld syonovial MSCs into the normal disc. Macroscopic views of interbertebral discs and fluorescent microscopic views of nucleus pulposus are shown. **(b) **Fluorescent microscopic views for GFP and DiI synovial MSCs. Nucleus pulposus was aspirated in both groups, and synovial MSCs were intradiscally injected into the MSC group.

DiI labeled synovial MSCs could be detected in the nucleus pulposus one day after intradiscal injection of the cells into the normal disc (Figure 2a). After aspiration of nucleus pulposus and injection of labeled synovial MSCs, DiI or GFP positive cells could be observed at 2, 8, and 24 weeks (Figure 2b).

### Imaging analyses for discs

**Figure 3:**
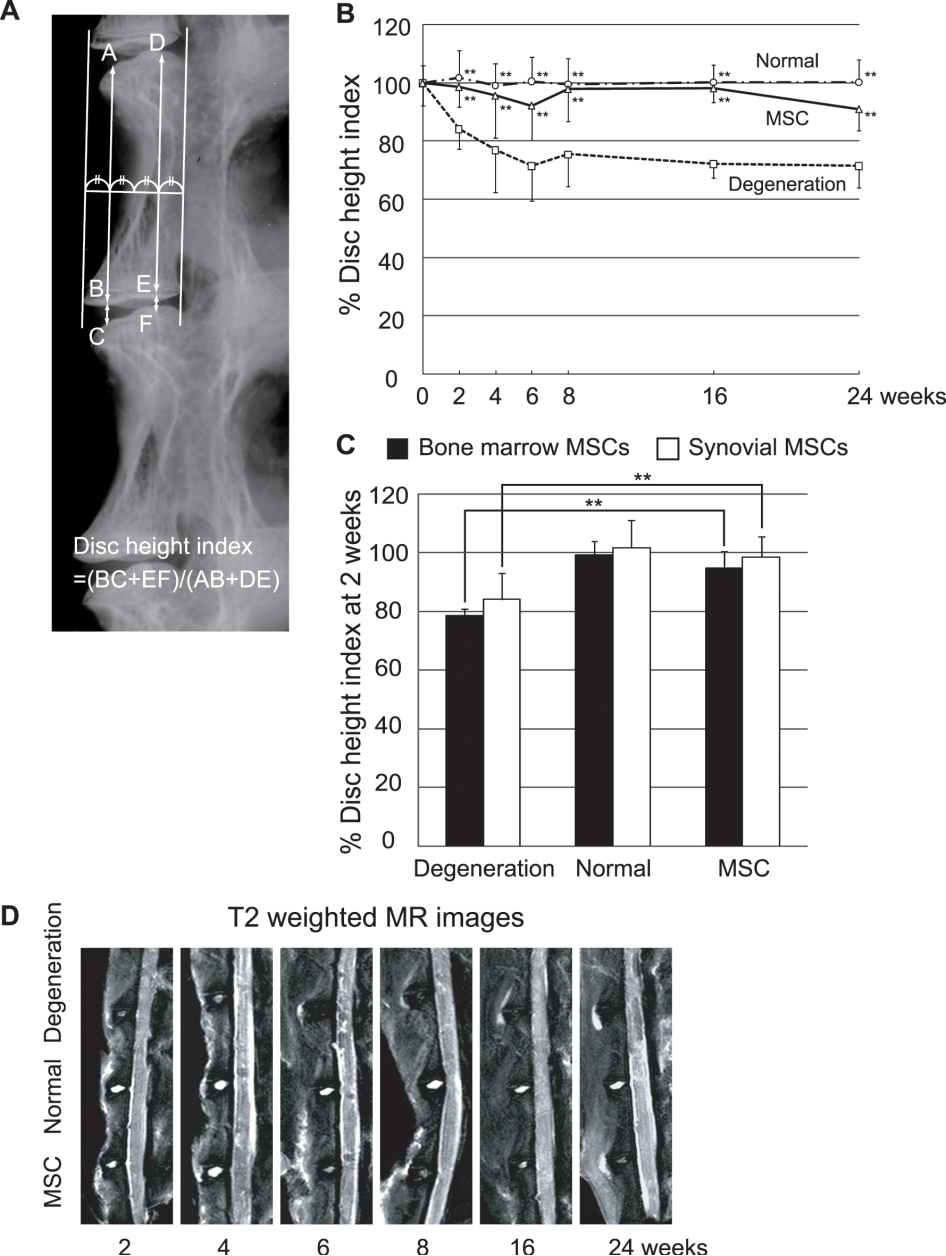
**Intradiscally injected MSCs maintain disc height**. **(a) **X-ray image of normal rabbit spine for measurement of disc height index. **(b) **Sequential changes of disc height index after transplantation of synovial MSCs. Average percentages of the value are shown with standard deviations. **P < 0.01 between the degeneration group and the normal group or the MSC group (*n *= 10 at each time point) by two-factor ANOVA and Turkey-Kramer post-hoc test. **(c) **Disc height index at two weeks after transplantation of bone marrow or synovial MSCs. Average percentages of values with standard deviations. **P < 0.01 between the bone marrow or synovial MSC group and the degeneration group (*n *= 6 for each group). **(d) **Representative T2-weighted MR images of intervertebral discs at 2 to 24 weeks after operation.

The disc height index was defined as the ratio of disc height to vertebral body height by lateral radiographs of the spine (Figure 3a). The disc height index in the degeneration group decreased gradually and reached bottom at six weeks. The disc height index in the MSC group was comparable to that in the normal group up until 24 weeks. The disc height index in the MSC group was statistically higher than that in the degeneration group at two weeks and thereafter (Figure 3b).

We also used injections of bone marrow MSCs instead of synovial MSCs, and compared the disc height index in both groups at two weeks. There was no significant difference of the disc height index between the bone marrow MSC group and the synovial MSC group, though the disc height index in each MSC group was significantly higher than that in each degeneration group (Figure 3c).

T2-weighted MR images showed that the signal intensity of nucleus pulposus in the degeneration group considerably decreased at two weeks and thereafter. Contrarily, the signal intensity of nucleus pulposus in the MSC group remained high comparable with that in the normal group at two and four weeks. Though the intensity in the MSC group gradually reduced after six weeks, it remained higher than that in the degeneration group up until 24 weeks (Figure 3d).

### Histological analysis

**Figure 4:**
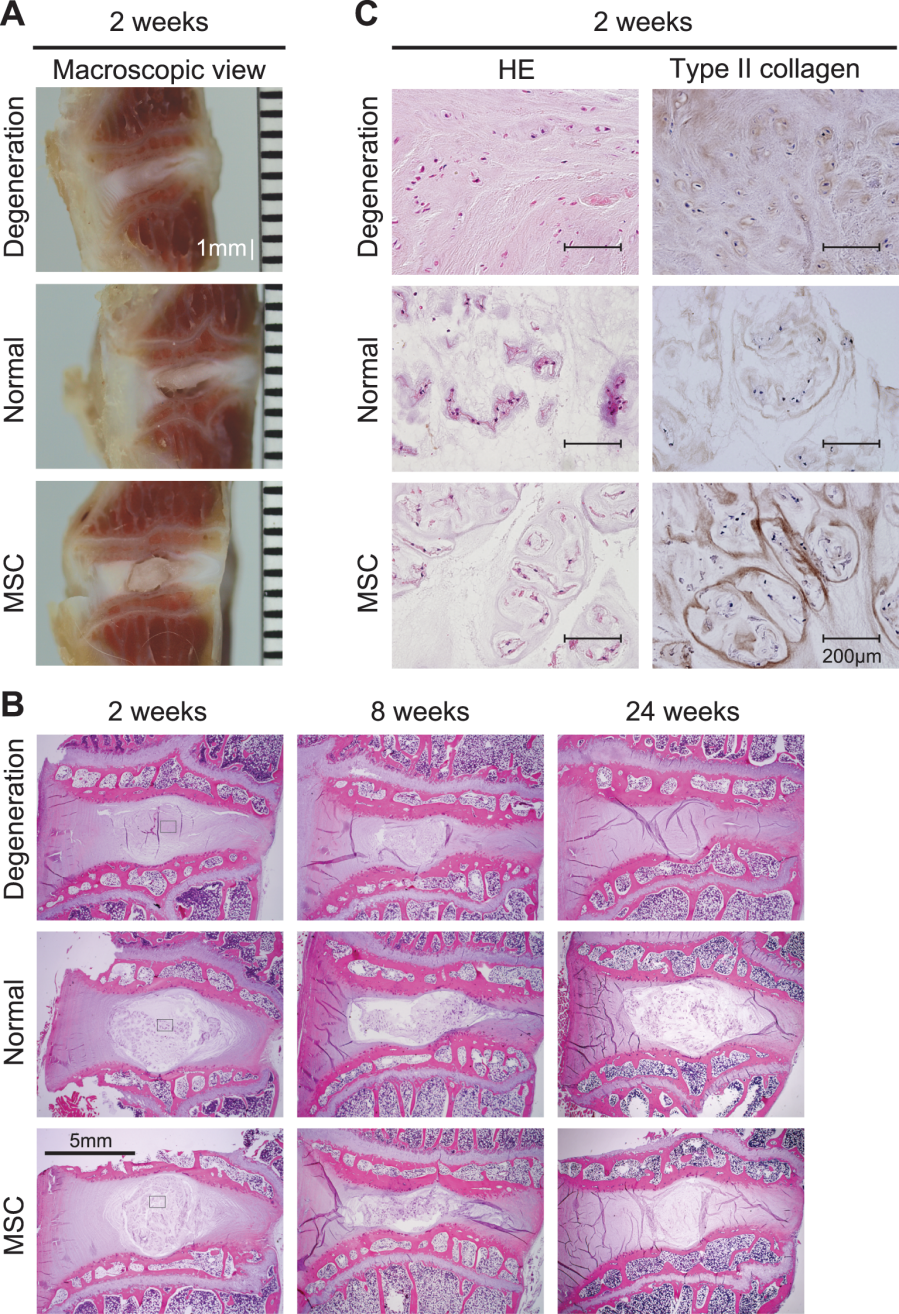
**Intradiscally injected MSCs maintain microstructure of nucleus pulpous**. **(a) **Macroscopic views of the sagittal section of intervertebral discs at two weeks after operation. **(b) **Sagittal sections with Hematoxylin-Eosin (HE) staining after operation. **(c) **Higher magnification of the framed area with type II collagen immunostaining.

According to macroscopic views of the sagittal section of intervertebral discs at 2 weeks after operation, in the degeneration group, the nucleus pulposus tissue volume was much less than that in the normal group (Figure 4a). In the MSC group, the nucleus pulposus was clearly observed.

In low magnified histologies, in the degeneration group, the nucleus pulposus could hardly be seen at two weeks and thereafter (Figure 4b). In the MSC group, the nucleus pulposus looked comparable to a normal one at two and eight weeks, and it was still visible at 24 weeks.

In high magnified histologies at two weeks, in the degeneration group, the nucleus pulposus was replaced with fibrous tissue (Figure 4c). In the MSC group, the nucleus pulposus consisted of sparse cells surrounded with matrix and looked similar to that of the normal group. Interestingly, type II collagen expression in the MSC group was higher than that in the normal group.

### Co-culture of human synovial MSCs and rat nucleus pulposus cells

**Figure 5:**
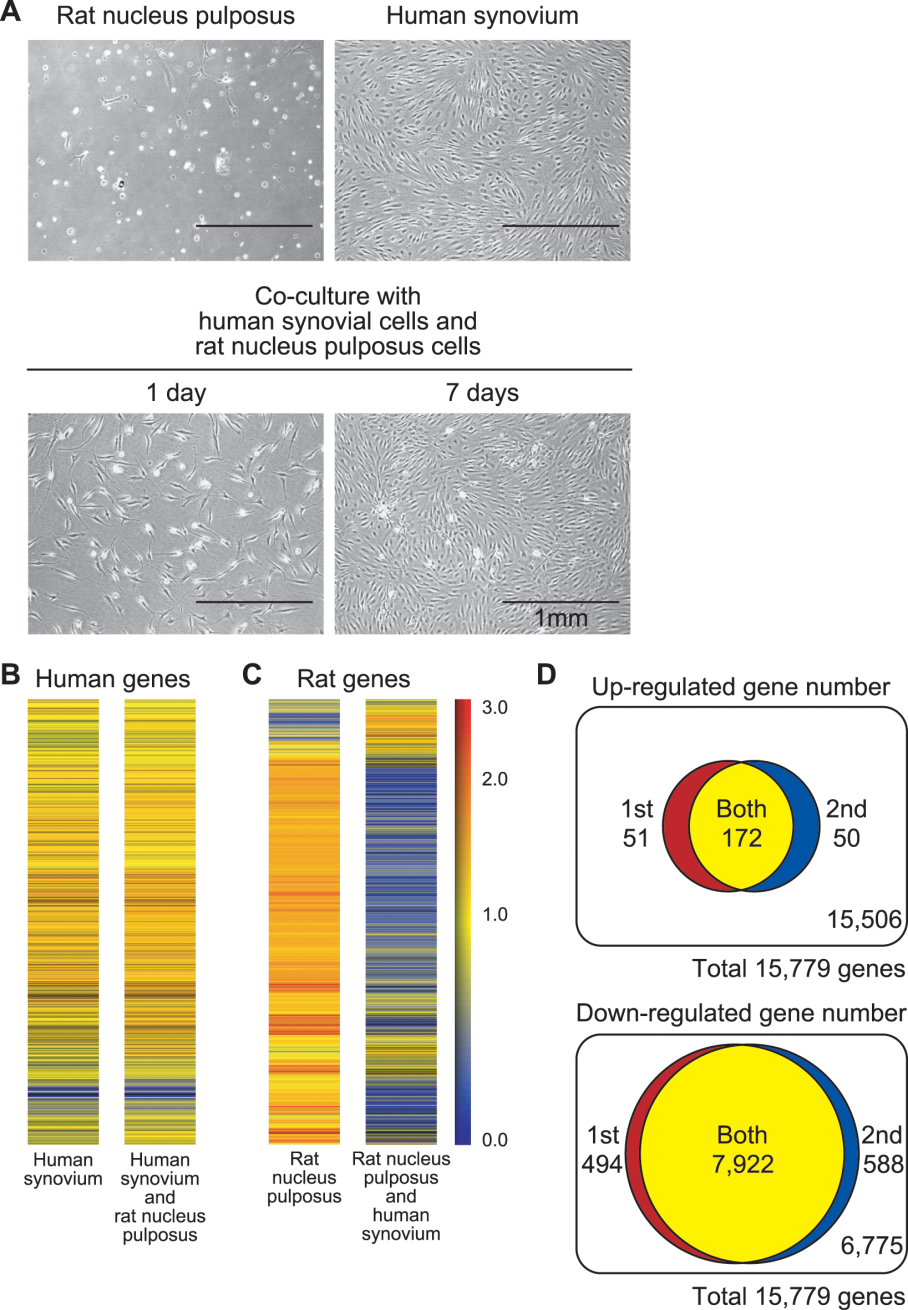
**Synovial MSCs affect gene profile of nucleus pulposus cells in co-culture system**. **(a) **Morphology of mono-culture of rat nucleus pulposus cells and human synovial MSCs at seven days, and co-culture of rat nucleus pulposus cells with human synovial MSCs at one and seven days. **(b) **Human gene profile of human synovial MSCs in mono-culture and in co-culture with rat nucleus pulposus cells. **(c) **Rat gene profile of rat nucleus pulposus cells in mono-culture and in co-culture with human MSCs. **(d) **Number of altered rat genes seven days after co-culture of rat nucleus pulposus cells with human synovial cells by duplicate of microarray analyses.

To investigate interaction between synovial MSCs and nucleus pulposus cells, human synovial MSCs and rat nucleus pulposus cells were co-cultured. Typical rat nucleus pulposus cells attached to the culture dish were bright and round (Figure 5a). Human synovial MSCs were spindle-shaped. Though a similar number of rat nucleus pulposus cells and human synovial MSCs were co-cultured, only human synovial MSCs appeared to increase in number at seven days (Figure 5a). Human microarray showed that the gene profile of human MSCs cultured alone was similar to that of human MSCs co-cultured with rat nucleus pulposus cells (Figure 5b). Contrarily, rat microarray demonstrated that the gene profile of rat nucleus pulposus cells was widely different from that of rat nucleus pulposus cells co-cultured with human MSCs (Figure 5c).

We further analyzed the gene profile of rat microarray. Among 31,099 transcripts, we first picked up 15,779 genes whose values were more than 50 and judged to be "present." Then, we selected rat genes whose expression value were two-fold higher or lower in co-culture than in mono-culture of rat nucleus pulposus cells. Two independent microarray analyses demonstrated 172 up-regulated genes and 7,922 down-regulated genes in common between the first and the second analysis (Figure 5d). Approximately 80% of the up-regulated genes and 90% of the down-regulated genes were overlapped as shown by the first and second microarray analyses, indicating reproducible results.

We next focused on the genes related to extracellular matrix, and the genes which possibly affect the extracellular matrix. We found five genes related to collagen, six genes related to proteoglycan, three genes related to tissue inhibitor of metalloproteinase (TIMP), seven genes related to matrix metalloproteinase (MMP), 10 genes related to interleukin (IL), and six genes related to tumor necrosis factor (TNF). Among these genes, up- and down- regulated genes two-fold or higher in co-culture are listed in Table 1. In collagens and proteoglycans, Col2A1, a principal component of nucleus pulposus, and Chondroitin sulfate proteoglycan 2, a member of the hyaluronan-binding proteoglycan family, were significantly up-regulated, though Aggrecan 1, another principal component of nucleus pulposus, was stable and is not listed in Table 1. Other collagen and proteoglycan related genes were mostly down-regulated. In the TIMP family, TIMP-3 was markedly up-regulated, though TIMP-1 and -2 were down-regulated. All the MMP genes listed on the microarray, especially MMP-2, -3, and -13, were down-regulated significantly. All inflammatory cytokine-related genes were also down-regulated. These data indicate that cartilage catabolic factors were suppressed and anabolic factors were enhanced, consequently contributing to the prevention of intervertebral disc degeneration.

**Table 1:** Rat genes up- and down- regulated two-fold or higher in nucleus pulposus cells co-cultured with human synovial MSCs

Genes	Genbank	Fold change
Collagens		
Col2a1	[Genbank:AF305418]	10.5
Col1a1	[Genbank:Z78279]	-2.3
Col5a1	[Genbank:NM_134452]	-7.1
Col5a3	[Genbank:NM_021760]	-25.3
Proteoglycans		
Chondroitin sulfate proteoglycan 2	[Genbank:AF084544]	2.9
Biglycan	[Genbank:NM_017087]	-7.6
Glypican 1	[Genbank:NM_030828]	-13.4
Lumican	[Genbank:NM_031050]	-16.1
Chondroitin sulfate proteoglycan 4	[Genbank:NM_031022]	-18.1
Tissue inhibitor of metalloproteinases		
TIMP-3	[Genbank:NM_012886]	27.3
TIMP-1	[Genbank:NM_053819]	-2.1
TIMP-2	[Genbank:NM_021989]	-4.7
Matrix metalloproteinases		
MMP-23	[Genbank:NM_053606]	-2.0
MMP-16	[Genbank:NM_080776]	-3.0
MMP-11	[Genbank:NM_012980]	-3.7
MMP-14	[Genbank:X83537]	-6.0
MMP-3	[Genbank:NM_133523]	-44.5
MMP-13	[Genbank:M60616]	-59.7
MMP-2	[Genbank:U65656]	-82.7
Interleukin related		
Nuclear factor, IL-3 regulated	[Genbank:NM_053727]	-2.3
IL-15	[Genbank:AF015718]	-3.5
IL-6 signal transducer	[Genbank:AI171807]	-7.4
IL-11 receptor, alpha chain 1	[Genbank:AF347936]	-9.3
Tumor necrosis factor related		
TNF receptor superfamily, member 1a (TNFRSF1A)	[Genbank:NM_013091]	-2.0
TNF-α converting enzyme	[Genbank:NM_020306]	-2.6
TNF-α induced protein 6 (TSG-6)	[Genbank:AF159103]	-5.4
Type 1 TNF receptor shedding aminopeptidase regulator	[Genbank:NM_030836]	-9.0
TNF receptor superfamily, member 6 (TNFRSF6)	[Genbank:BE108106]	-12.5

A total of 15,779 rat genes consistent with the quality criteria, genes in collagens, proteoglycans, tissue inhibitor of metalloproteinases, matrix metalloproteinases, interleukin-, and tumor necrosis factor-related genes are listed. IL, interleukin; MMP, matrix metalloproteinase; TIMP, tissue inhibitor of metalloproteinase; TNF, tumor necrosis factor.

## Discussion

In this study, we demonstrate that intradiscal injection of synovial MSCs prevented intervertebral disc degeneration in rabbits up until 24 weeks. Several reports have shown differentiation of bone marrow MSCs toward a nucleus pulposus-like phenotype *in vitro *[21-23] and the regenerative effects of bone marrow MSCs after intradiscal injection [8-10]. To the best of our knowledge, only one paper has shown *in vitro *differentiation of synovial MSCs into nucleus pulposus in which synovial MSCs and nucleus pulposus cells were co-cultured [24]. Ours is the first report demonstrating the effectiveness of intradiscal transplantation of synovial MSC in rabbit intervertebral disc degeneration model.

For tracking the transplanted cells, we used DiI and GFP systems. DiI is a popular dye, highly fluorescent and photostable when incorporated into lipid membrane [25,26]. It exhibits low cell toxicity [27], and retains its fluorescence for a long time in specific situations. However, there is some criticism in the use of DiI. The emission of DiI fluorescence decreases every time cells divide. If DiI leaches out of dying cells, it may be doubtful whether DiI fluorescence indicates living transplanted cells or not. In our case, if the dye had leaked from the injected MSCs, they would not have emitted significant fluorescence in the extracellular matrix of nucleus pulposus, because DiI almost never emits fluorescent in aqueous solutions. When leaked DiI transfers between intact membranes, DiI is usually negligible [28]. To verify the results of tracking cells, we also transplanted synovial MSCs derived from a GFP transgenic rabbit. We could observe GFP-positive and/or DiI labeled cells at the nucleus pulposus 24 weeks after transplantation, which demonstrates that transplanted MSCs survived for some time in the nucleus pulposus.

According to X-ray and histological analyses, the effect of MSCs could be observed at 24 weeks. Immunohistological analyses demonstrated that the amount of type II collagen in the nucleus pulposus was higher in the MSC group than even in the normal group at two weeks. Generally, type II collagen functions as a frame work in cartilage tissues [1,29]. These findings indicate that transplantation of MSCs induced a higher amount of type II collagen, which acted as a frame work in the nucleus pulposus, which resulted in the maintenance of disc height and histological features.

On the other hand, based on MR imaging, the effect of MSCs already decreased at six weeks. High signal intensity of T2 weighted MR imaging reflects the amount of water in the nucleus pulposus. Possibly, transplanted MSCs promoted synthesis of proteoglycan, in which negative charged sulfate held water. In our study, the ability of MSCs to maintain water was reduced at six weeks. During the degeneration of intervertebral disc in humans, reduction of water content in the nucleus pulposus by MR imaging precedes a decrease of disc height as shown by X-ray [30,31].

In this model, transplantation of MSCs into the intervertebral disc delayed progression of degeneration, but its effect was not maintained due to MRI imaging. This result is different from that of previous studies demonstrating regeneration of articular cartilage. After synovial MSCs were transplanted into full thickness defect of articular cartilage, cartilage matrix was filled in the defect. Then the border between the bone and cartilage was moved, and finally the thickness of the regenerated cartilage became similar to that of the adjacent native cartilage. The regenerated cartilage was maintained at least for six months [14,32,33]. In our current study, though extracellular matrix was reproduced by synovial MSC in the nucleus pulposus, the nucleus pulposus never thickened more than the normal one. These differences were thought to be caused by environmental differences. Intervertebral disc is a thicker avascular tissue than articular cartilage, and it is not surrounded by joint fluid or synovium. A severer environment around the intervertebral disc may reduce the regenerative effects of MSCs.

Species specific microarray analyses in our co-culture experiment revealed that nucleus pulposus cells dramatically changed their gene profile by interaction with synovial MSCs. Contrarily, synovial MSCs did not change their gene profile in co-culture with nucleus pulposus cells. These results demonstrate that synovial MSCs influenced nucleus pulposus cells, but nucleus pulposus cells did not affect synovial MSCs.

Co-culture of synovial MSCs increased Col2a1 expression more than 10-fold in nucleus pulposus cells. An *in vivo *study demonstrated that transplantation of synovial MSCs enhanced type II collagen expression in the nucleus pulposus immunohistologically. We could not detect type II collagen expression around labeled MSCs. It is well known that turnover of cartilage collagen is very slow [34]. These findings indicate that synovial MSCs promoted the remaining nucleus pulposus to synthesize type II collagen.

Among genes for proteoglycans, chondroitin sulfate proteoglycan 2 expression increased to about 3-fold in nucleus pulposus cells with co-culture of synovial MSCs. Chondroitin sulfate proteoglycan 2, known as versican, is one of the principal components of nucleus pulposus, and its expression is higher in nucleus pulposus than in articular cartilage [35,36]. A possible higher expression of chondroitin sulfate proteoglycan 2 in nucleus pulposus cells induced the holding of water, resulting in improvements of MR imaging in an *in vivo *study.

A number of studies have noted that degradative enzymes and inflammatory cytokines are highly produced by nucleus pulposus cells in degenerate intervertebral discs [37,38]. According to our microarray analyses, all MMP genes examined were down-regulated in nucleus pulposus cells with co-culture of synovial MSCs. In particular, MMP-2, -3, -13 were highly down-regulated, and their inhibitor TIMP-3 expression increased more than 27-fold. It is known that TIMP-3 particularly inhibits aggrecanases [38] and that MMP-13, known as collagenase-3, has a strong effect on type II collagen [39]. MMP-2 was also found in degenerated discs and had a highly significant correlation with age and histological alterations of intervertebral discs [40]. Suppression of MMPs results in an increase of type II collagen expression *in vivo*.

All interleukin related and tumor necrosis factor related genes listed were down-regulated. All of them have inflammatory effects. Inhibition of inflammatory cytokine-related genes could induce the suppression of MMPs [39,41] which resulted in the preventive effects of intervertebral disc degeneration.

For clinical application, two things should be considered. First, species differences will affect the results. We used rabbits in which nucleus pulposus contained more notochordal cells than found in adult humans. Notochordal cells have a higher regenerative potential than other cells in the nucleus pulposus [42,43]. Second, the choice of intervertebral disc degeneration model should be considered. In this study, disc degeneration was induced by aspiration of nucleus pulposus tissues. This model is caused traumatically and does not mimic human age-dependent intervertebral disc degeneration. However, a nucleus pulposus aspiration model is useful in that nucleus pulposus promptly decreases after aspiration, and this method could provide rapid and stable induction of disc degeneration.

The preventive effect of synovial MSCs was similar to that of bone marrow MSCs in our study. Though it may be thought that the availability of synovium in human is lower than that of bone marrow, it really is not. We are currently conducting a clinical study of transplantation of autologous synovial MSCs into cartilage defect of the knee. Synovial tissue can be harvested from the knee joint under local anesthesia without arthroscopy in clinical practice. Synovial MSCs can be expanded faster and greater with autologous human serum than bone marrow MSCs [15], which is an advantage of synovial MSCs. Synovial MSCs are a potential cell source for intervertebral disc regeneration therapy as well as bone marrow MSCs.

**Figure 6:**
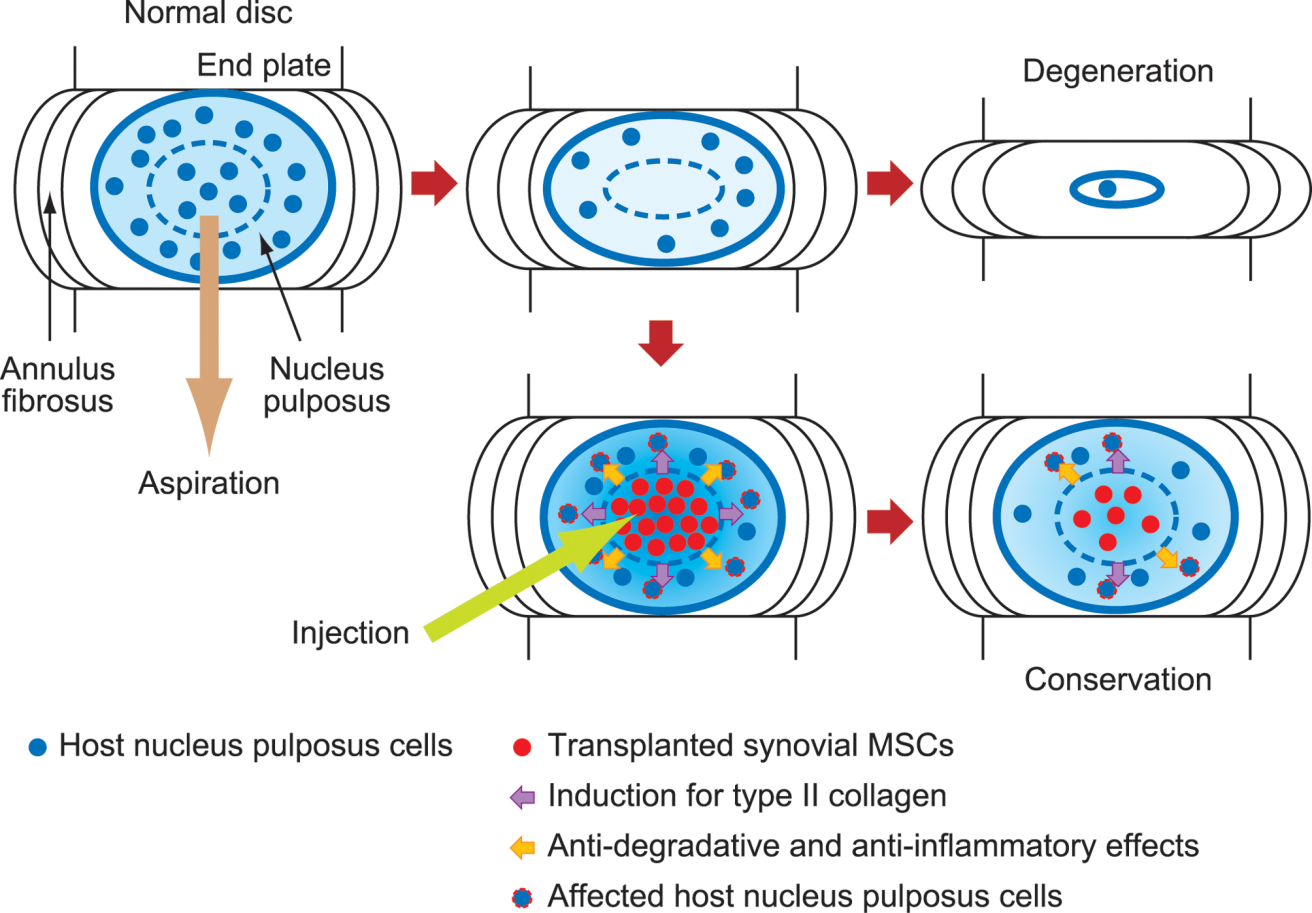
**Possible mechanism of prevention for intervertebral disc degeneration by intradiscal transplantation of synovial MSCs**. After aspiration of the nucleus pulposus, intervertebral disc space rapidly decreases. Synovial MSCs injected into the nucleus pulposus space promote synthesis of type II collagen for the remaining nucleus pulposus cells. Also, synovial MSCs affected the remaining nucleus pulposus cells by inhibiting expressions of degradative enzymes and inflammatory cytokines, resulting in maintaining the structure of the vertebral disc.

We summarize a possible mechanism of prevention for intervertebral disc degeneration by intradiscal transplantation of synovial MSCs shown in Figure 6. After aspiration of the nucleus pulposus, intervertebral disc space rapidly decreases. Synovial MSCs injected into the nucleus pulposus space promoted the remaining nucleus pulposus cells to synthesize type II collagen. Also, synovial MSCs affected the remaining nucleus pulposus cells by inhibiting their expressions of degradative enzymes and inflammatory cytokines, resulting in maintaining the structure of the intervertebral disc.

## Conclusions

Intradiscal transplantation of synovial MSCs prevented intervertebral disc degeneration *in vivo*. Co-culture assay *in vitro *revealed that nucleus pulposus cells dramatically changed their gene profile by interaction with synovial MSCs to inhibit expressions of the genes for degradative enzymes and inflammatory cytokines.

## Abbreviations

αMEM: α-minimal essential medium; DHI: disc height index; EDTA: ethylenediaminetetraacetate; FBS: fetal bovine serum; FOV: field of view; GFP: green fluorescent protein; IL: interleukin; IVT: *in vitro *transcription; MMP: matrix metalloproteinase; MRI: magnetic resonance imaging; MSC: mesenchymal stem cell; PBS: phosphate-buffered saline; TE: echo time; TIMP: tissue inhibitor of metalloproteinase; TNF: tumor necrosis factor; TR: repetition time.

## Competing interests

The authors declare that they have no competing interests.

## Authors' contributions

TMi participated in the design of the study, carried out the animal experiments, analyzed the results, and drafted the manuscript. TMu participated in the design of the study and provided the administrative and financial support. TT carried out the MR imaging. KM and HS carried out the microarray and participated in the evaluation of the results. KT participated in the design of the study and provided the financial support. IS participated in the design of the study, provided the financial support, and completed the final manuscript. All authors read and approved the final manuscript.

## References

[B1] Humzah MD Soames RW Human intervertebral disc: structure and function Anat Rec 1988 220 337 356 10.1002/ar.1092200402 3289416

[B2] Reuler J Low back pain West J Med 1985 143 259 265 2930949 PMC1306303

[B3] Deyo R Weinstein J Low back pain N Engl J Med 2001 344 363 370 10.1056/NEJM200102013440508 11172169

[B4] Walsh A Bradford D Lotz J *In vivo *growth factor treatment of degenerated intervertebral discs Spine (Phila Pa 1976) 2004 29 156 163 14722406 10.1097/01.BRS.0000107231.67854.9F

[B5] Masuda K Imai Y Okuma M Muehleman C Nakagawa K Akeda K Thonar E Andersson G An HS Osteogenic protein-1 injection into a degenerated disc induces the restoration of disc height and structural changes in the rabbit anular puncture model Spine 2006 31 742 754 10.1097/01.brs.0000206358.66412.7b 16582847

[B6] Cui M Wan Y Anderson D Shen F Leo B Laurencin C Balian G Li X Mouse growth and differentiation factor-5 protein and DNA therapy potentiates intervertebral disc cell aggregation and chondrogenic gene expression Spine J 2008 8 287 295 10.1016/j.spinee.2007.05.012 17974491

[B7] Nomura T Mochida J Okuma M Nishimura K Sakabe K Nucleus pulposus allograft retards intervertebral disc degeneration Clin Orthop Relat Res 2001 94 101 10.1097/00003086-200108000-00015 11501830

[B8] Crevensten G Walsh A Ananthakrishnan D Page P Wahba G Lotz J Berven S Intervertebral disc cell therapy for regeneration: mesenchymal stem cell implantation in rat intervertebral discs Ann Biomed Eng 2004 32 430 434 10.1023/B:ABME.0000017545.84833.7c 15095817

[B9] Zhang YG Guo X Xu P Kang LL Li J Bone mesenchymal stem cells transplanted into rabbit intervertebral discs can increase proteoglycans Clin Orthop Relat Res 2005 219 226 10.1097/01.blo.0000146534.31120.cf 15662327

[B10] Sakai D Mochida J Iwashina T Hiyama A Omi H Imai M Nakai T Ando K Hotta T Regenerative effects of transplanting mesenchymal stem cells embedded in atelocollagen to the degenerated intervertebral disc Biomaterials 2006 27 335 345 10.1016/j.biomaterials.2005.06.038 16112726

[B11] Sakaguchi Y Sekiya I Yagishita K Muneta T Comparison of human stem cells derived from various mesenchymal tissues: superiority of synovium as a cell source Arthritis Rheum 2005 52 2521 2529 10.1002/art.21212 16052568

[B12] Mochizuki T Muneta T Sakaguchi Y Nimura A Yokoyama A Koga H Sekiya I Higher chondrogenic potential of fibrous synovium- and adipose synovium-derived cells compared with subcutaneous fat-derived cells: distinguishing properties of mesenchymal stem cells in humans Arthritis Rheum 2006 54 843 853 10.1002/art.21651 16508965

[B13] Yoshimura H Muneta T Nimura A Yokoyama A Koga H Sekiya I Comparison of rat mesenchymal stem cells derived from bone marrow, synovium, periosteum, adipose tissue, and muscle Cell Tissue Res 2007 327 449 462 10.1007/s00441-006-0308-z 17053900

[B14] Koga H Muneta T Nagase T Nimura A Ju Y Mochizuki T Sekiya I Comparison of mesenchymal tissues-derived stem cells for *in vivo *chondrogenesis: suitable conditions for cell therapy of cartilage defects in rabbit Cell Tissue Res 2008 333 207 215 10.1007/s00441-008-0633-5 18560897

[B15] Nimura A Muneta T Koga H Mochizuki T Suzuki K Makino H Umezawa A Sekiya I Increased proliferation of human synovial mesenchymal stem cells with autologous human serum: comparisons with bone marrow mesenchymal stem cells and with fetal bovine serum Arthritis Rheum 2008 58 501 510 10.1002/art.23219 18240254

[B16] Takahashi R Kuramochi T Aoyagi K Hashimoto S Miyoshi I Kasai N Hakamata Y Kobayashi E Ueda M Establishment and characterization of CAG/EGFP transgenic rabbit line Transgenic Res 2007 16 115 120 10.1007/s11248-006-9043-1 17103241

[B17] Lü D Shono Y Oda I Abumi K Kaneda K Effects of chondroitinase ABC and chymopapain on spinal motion segment biomechanics. An *in vivo *biomechanical, radiologic, and histologic canine study Spine (Phila Pa 1976) 1997 22 1828 1834 discussion 1834-1825 9280018 10.1097/00007632-199708150-00006

[B18] Kato A Homma T Batchelor J Hashimoto N Imai S Wakiguchi H Saito H Matsumoto K Interferon-alpha/beta receptor-mediated selective induction of a gene cluster by CpG oligodeoxynucleotide 2006 BMC Immunol 2003 4 8 10.1186/1471-2172-4-8 12887736 PMC183869

[B19] Gene Expression Omnibus http://www.ncbi.nlm.nih.gov/projects/geo/

[B20] Dominici M Le Blanc K Mueller I Slaper-Cortenbach I Marini F Krause D Deans R Keating A Prockop D Horwitz E Minimal criteria for defining multipotent mesenchymal stromal cells. The International Society for Cellular Therapy position statement Cytotherapy 2006 8 315 317 10.1080/14653240600855905 16923606

[B21] Risbud MV Albert TJ Guttapalli A Vresilovic EJ Hillibrand AS Vaccaro AR Shapiro IM Differentiation of mesenchymal stem cells towards a nucleus pulposus-like phenotype *in vitro*: implications for cell-based transplantation therapy Spine 2004 29 2627 2632 10.1097/01.brs.0000146462.92171.7f 15564911

[B22] Steck E Bertram H Abel R Chen B Winter A Richter W Induction of intervertebral disc-like cells from adult mesenchymal stem cells Stem Cells 2005 23 403 411 10.1634/stemcells.2004-0107 15749935

[B23] Richardson S Hughes N Hunt J Freemont A Hoyland J Human mesenchymal stem cell differentiation to NP-like cells in chitosan-glycerophosphate hydrogels Biomaterials 2008 29 85 93 10.1016/j.biomaterials.2007.09.018 17920676

[B24] Chen S Emery S Pei M Coculture of synovium-derived stem cells and nucleus pulposus cells in serum-free defined medium with supplementation of transforming growth factor-beta1: a potential application of tissue-specific stem cells in disc regeneration Spine (Phila Pa 1976) 2009 34 1272 1280 19455002 10.1097/BRS.0b013e3181a2b347

[B25] McCauley D Bronner-Fraser M Neural crest contributions to the lamprey head Development 2003 130 2317 2327 10.1242/dev.00451 12702647

[B26] Mothe A Tator C Proliferation, migration, and differentiation of endogenous ependymal region stem/progenitor cells following minimal spinal cord injury in the adult rat Neuroscience 2005 131 177 187 10.1016/j.neuroscience.2004.10.011 15680701

[B27] Crawford J Braunwald N Toxicity in vital fluorescence microscopy: effect of dimethylsulfoxide, rhodamine-123, and DiI-low density lipoprotein on fibroblast growth *in vitro* In Vitro Cell Dev Biol 1991 27A 633 638 10.1007/BF02631106 1917780

[B28] Andrade W Seabrook T Johnston M Hay J The use of the lipophilic fluorochrome CM-DiI for tracking the migration of lymphocytes J Immunol Methods 1996 194 181 189 10.1016/0022-1759(96)00083-X 8765171

[B29] Nerlich A Boos N Wiest I Aebi M Immunolocalization of major interstitial collagen types in human lumbar intervertebral discs of various ages Virchows Arch 1998 432 67 76 10.1007/s004280050136 9463590

[B30] Berlemann U Gries N Moore R The relationship between height, shape and histological changes in early degeneration of the lower lumbar discs Eur Spine J 1998 7 212 217 10.1007/s005860050058 9684954 PMC3611258

[B31] Kim K Yoon S Li J Park J Hutton W Disc degeneration in the rabbit: a biochemical and radiological comparison between four disc injury models Spine (Phila Pa 1976) 2005 30 33 37 15626978 10.1097/01.brs.0000149191.02304.9b

[B32] Koga H Muneta T Ju YJ Nagase T Nimura A Mochizuki T Ichinose S von der Mark K Sekiya I Synovial stem cells are regionally specified according to local microenvironments after implantation for cartilage regeneration Stem Cells 2007 25 689 696 10.1634/stemcells.2006-0281 17138960

[B33] Koga H Shimaya M Muneta T Nimura A Morito T Hayashi M Suzuki S Ju Y Mochizuki T Sekiya I Local adherent technique for transplanting mesenchymal stem cells as a potential treatment of cartilage defect Arthritis Res Ther 2008 10 R84 10.1186/ar2460 18664254 PMC2575632

[B34] Verzijl N DeGroot J Thorpe S Bank R Shaw J Lyons T Bijlsma J Lafeber F Baynes J TeKoppele J Effect of collagen turnover on the accumulation of advanced glycation end products J Biol Chem 2000 275 39027 39031 10.1074/jbc.M006700200 10976109

[B35] Sztrolovics R Grover J Cs-Szabo G Shi S Zhang Y Mort J Roughley P The characterization of versican and its message in human articular cartilage and intervertebral disc J Orthop Res 2002 20 257 266 10.1016/S0736-0266(01)00110-3 11918305

[B36] Roughley P Biology of intervertebral disc aging and degeneration: involvement of the extracellular matrix Spine (Phila Pa 1976) 2004 29 2691 2699 15564918 10.1097/01.brs.0000146101.53784.b1

[B37] Roberts S Caterson B Menage J Evans E Jaffray D Eisenstein S Matrix metalloproteinases and aggrecanase: their role in disorders of the human intervertebral disc Spine (Phila Pa 1976) 2000 25 3005 3013 11145811 10.1097/00007632-200012010-00007

[B38] Le Maitre C Freemont A Hoyland J Localization of degradative enzymes and their inhibitors in the degenerate human intervertebral disc J Pathol 2004 204 47 54 10.1002/path.1608 15307137

[B39] Le Maitre C Freemont A Hoyland J The role of interleukin-1 in the pathogenesis of human intervertebral disc degeneration Arthritis Res Ther 2005 7 R732 745 10.1186/ar1732 15987475 PMC1175026

[B40] Weiler C Nerlich A Zipperer J Bachmeier B Boos N 2002 SSE Award Competition in Basic Science: expression of major matrix metalloproteinases is associated with intervertebral disc degradation and resorption Eur Spine J 2002 11 308 320 10.1007/s00586-002-0472-0 12193991 PMC3610483

[B41] Séguin C Pilliar R Madri J Kandel R TNF-alpha induces MMP2 gelatinase activity and MT1-MMP expression in an *in vitro *model of nucleus pulposus tissue degeneration Spine (Phila Pa 1976) 2008 33 356 365 18277865 10.1097/BRS.0b013e3181642a5e

[B42] Hunter C Matyas J Duncan N The notochordal cell in the nucleus pulposus: a review in the context of tissue engineering Tissue Eng 2003 9 667 677 10.1089/107632703768247368 13678445

[B43] Miyazaki T Kobayashi S Takeno K Meir A Urban J Baba H A phenotypic comparison of proteoglycan production of intervertebral disc cells isolated from rats, rabbits, and bovine tails; which animal model is most suitable to study tissue engineering and biological repair of human disc disorders? Tissue Eng Part A 2009 15 3835 3846 10.1089/ten.tea.2009.0250 19681728

